# Social inequalities in BMI trajectories: 8-year follow-up of the Pró-Saúde study in Rio de Janeiro, Brazil

**DOI:** 10.1017/S1368980015001032

**Published:** 2015-04-21

**Authors:** Dóra Chor, Valeska Andreozzi, Maria JM Fonseca, Letícia O Cardoso, Sherman A James, Claudia S Lopes, Eduardo Faerstein

**Affiliations:** 1 National School Public Health, Oswaldo Cruz Foundation, Rua Leopoldo Bulhões 1480, Room 813, Rio de Janeiro, RJ, CEP 21041-210, Brazil; 2 Centre of Statistics and Applications, University of Lisbon, Lisbon, Portugal; 3 Faculty of Medical Sciences, New University of Lisbon, Lisbon, Portugal; 4 Department of Epidemiology, Rollins School of Public Health, Emory University, Atlanta, GA, USA; 5 Institute of Social Medicine, Rio de Janeiro State University, Rio de Janeiro, Brazil

**Keywords:** Weight gain, Health status disparities, Race/ethnicity, Cohort studies

## Abstract

**Objective:**

In a cohort of government employees in Rio de Janeiro, Brazil, we investigated prospectively, sex-specific associations between education and BMI trajectories and their potential effect modification by race.

**Design:**

Of the 4030 participants in Phase 1 (1999), 3253 (81 %) participated in Phase 2 (2003) and 3058 (76 %) participated in Phase 3 (2006). Education was categorized as elementary, high school or college graduate. Study participants self-identified as White, Black or *Pardo*. BMI was calculated from measured weight and height. BMI trajectories were modelled using a generalized additive regression model with mixed effects (GAMM).

**Setting:**

The Pro-Saúde Study, a longitudinal investigation of social determinants of health.

**Subjects:**

Women (*n* 1441) and men (*n* 1127) who participated in the three phases of data collection and had complete information for all study variables.

**Results:**

Women and men with less than high school, or only a high school education, gained approximately 1 kg/m^2^ more than college graduates (women: 1·06 kg/m^2^ (*P*<0·001) and 1·06 kg/m^2^ (*P*<0·001), respectively; men: 1·04 kg/m^2^ (*P=*0·013) and 1·01 kg/m^2^ (*P*=0·277), respectively). For women only, race was independently associated with weight gain. Women identifying as *Pardo* or Black gained 1·03 kg/m^2^ (*P*=0·01) and 1·02 kg/m^2^ (*P*=0·10), respectively, more than Whites. No effect modification by race was observed for either men or women.

**Conclusions:**

While both lower education and darker race were associated with greater weight gain, gender similarities and differences were observed in these associations. The relationship between weight gain and different indicators of social status are therefore complex and require careful consideration when addressing the obesity epidemic.

Obesity is a global epidemic, the origin of which lies in the complexity of human behaviour with well-recognized social determinants, although the exact mechanisms involved are not fully understood. These determinants – contextual, behavioural and biological – have been studied mostly in isolation, which ignores their inherent interrelationships. Obesity is a challenge to public health, which aims to control the condition and reduce inequalities within and across populations^(^ ^1^ ^,^ ^2^ ^)^. Studies conducted in more than forty countries between 1990 and 2010 suggest that two billion people are overweight or obese^(^ ^3^ ^)^.

In Brazil, the prevalence of excess weight – overweight and obesity – increased from 19 % (men) and 29 % (women) in 1974/75 to 50 % and 48 %, respectively, in 2008/09. The inverse association consistently found between socio-economic status and overweight and obesity in other countries, particularly among women, was also observed in Brazil during the last 20 years^(^ ^4^ ^–^ ^7^ ^)^. Between 1975 and 1989, obesity increased in most regions of the country among population groups with higher levels of education. Between 1989 and 1997, however, this trend was reversed. The largest increase occurred among those who did not attend school, and stabilized or even declined among women with higher levels of education^(^ ^8^ ^)^. More recent results (between 2006 and 2009) have revealed a new trend, in which the increasing prevalence of obesity is similar in men of different educational levels, but among women inequality has widened. Obesity increased from 15·2 % to 18·2 % among those with up to 8 years of education, and only from 7·5 % to 8·4 % in those with 12 or more years of education^(^ ^9^ ^)^.

The increasing prevalence of obesity has particularly serious medical consequences for the poorest populations, including ethnic and racial groups historically suffering discrimination^(^ ^9^ ^)^. In the USA, there is evidence that socio-economic inequalities in health are strongly shaped by race^(^ ^10^ ^)^. In Brazil, the combined influence of socio-economic indicators and race on health has been poorly studied^(^ ^11^ ^–^ ^14^ ^)^. We consider race to be a social construct, but one that has both real and wide-ranging consequences for population health and health disparities. This discussion is recent, and controversial, in Brazilian society.

Compared with Whites, Brazilian Blacks and *Pardo* (to mean ‘mixed’ race) are known to be systematically disadvantaged regarding educational attainment and job market positions. From 1960 through 2000, the latter groups were threefold more likely to be poor and illiterate than Whites. More recent data confirm such disadvantage in education and the job market^(^ ^15^ ^)^.

Our analyses are framed within an intersectionality context^(^ ^16^ ^)^. We postulate that education, race and gender, all relevant axes of social stratification, interact to influence weight trajectory through more proximal determinants of weight change. Education determines in several ways the possibility of adopting what is defined as healthy behaviours^(^ ^17^ ^)^. Social adversity, however, manifests itself differently according to race, adding to the chronic stress caused by discrimination^(^ ^18^ ^)^. Finally, it is known that men and women react differently to obesogenic contexts to which they are exposed^(^ ^19^ ^)^.

In Brazil, evidence for population trends of excess weight comes from cross-sectional studies^(^ ^7^ ^–^ ^9^ ^)^, which limits a full understanding of their social determinants. Considering that relationships between socio-economic indicators and race are distinct from those found in places where racial segregation has been legal, we investigated the association between education and BMI trends, as well as potential modifying effects of race, in a cohort of civil servants in Rio de Janeiro.

## Materials and methods

### Study design and population

The Pro-Saúde Study is a longitudinal investigation of social determinants of health among non-faculty civil servants at a large university in the state of Rio de Janeiro, Brazil. All employees except those relocating to another institution or who were on non-health leave were eligible to participate^(^ ^20^ ^)^. With a participation rate of 90·4 % among all eligible employees, 4030 participated in Phase 1 of the study (1999). Follow-up data collections were conducted in 2001 (Phase 2) and 2006–2007 (Phase 3). Phase 4 data collection began in 2011. Among the 4030 respondents who participated in Phase 1, 3253 (81 %) participated in Phase 2 and 3058 (76 %) in Phase 3.

We analysed data for those who participated in the three phases of data collection (3058 participants) and who also had complete information for all study variables. This totalled 2568 participants: 1441 women and 1127 men. We excluded sixty participants owing to lack of information about parental education and 120 due to missing data on race. Among other covariates, the percentage of missing data ranged from 2·6 % for marital status to 5·9 % for per capita family income, for a total of 490 exclusions due to missing data. The final distribution of study participants was as follows: 1441 women (of whom 1425, 1411 and 1419 were involved in Phases 1, 2 and 3, respectively) and 1127 men (of whom 1121, 1108 and 1101 were involved in the respective phases).

### Statement of ethics

This study was conducted according to the guidelines laid down in the Declaration of Helsinki and all procedures involving human subjects/patients were approved by the Ethics Committee of Rio de Janeiro State University. Written informed consent was obtained from all subjects/patients.

### Measures

In all study phases, participants’ weight and height were measured. Self-administered questionnaires were applied, including questions about sociodemographic characteristics, medical history, health-related behaviours and reported experiences of discrimination. Pre-tests and pilot studies with test–retest reliability studies were conducted in all phases of data collection and deemed adequate^(^ ^20^ ^)^.

BMI trends were the outcomes of interest. BMI was estimated by dividing the weight in kilograms by the square of height in metres. Weight was measured using an electronic scale with a maximum capacity of 150 kg and an accuracy of 50 g. Height was measured by following the techniques proposed by Lohman *et al*.^(^ ^21^ ^)^, using a tape measure affixed to a flat wall and a mobile set-square. Procedures were the same in all phases of data collection, following continuous training and with supervision for quality control.

The main variable of interest in the present study, formal education, was categorized into three levels: elementary, high school, and college or higher. Race was tested as an effect modifier because it is an additional indicator of social disadvantage and discrimination. In Brazil, a person’s ‘race’ is defined on the basis of his/her physical appearance (e.g. skin colour, hair texture, and conformation of nose and lips) rather than ancestral origin. This is quite different from racial categories used in the USA. Our study participants were asked to self-identify with one of the categories used by the Brazilian Census: White, *Pardo*, Black, Asian or indigenous. We excluded employees who self-identified as Asian and indigenous, because they represented less than 3 % (fifty-three Asian and thirty-two indigenous) of the total study population.

Other covariates were year of data collection, age, gender, marital status, per capita family income, smoking habits and parental education. The year of data collection (phase) was analysed as a categorical variable (1999, 2001 and 2006). Age was measured in years and centred on the average value measured in the first phase; marital status was classified as single, married and widow(er)/separated; per capita family income in minimum wages was calculated from the midpoint of the category of reported net income, divided by the number of people supported by this income. This value was then divided by the value of the minimum wage (roughly $US 141·00 in year 2000) at the time of data collection and grouped into three categories: <3 minimum wages, 3–6 minimum wages and >6 minimum wages. Three categories of smoking behaviour were created: never smoked, quit smoking (1 year or more) and currently smoke. The educational level of the parents was classified as not having attended school, or attained primary, high school or college education. When, for a given individual, parents’ levels of education were unequal, the highest level was used in the analysis. All independent variables, except for age, were collected at baseline.

### Statistical analysis

A generalized additive regression model with mixed effects (GAMM) was applied to model the repeated BMI measurements on the same participant^(^ ^22^ ^)^. GAMM, besides accounting for possible non-linearity of age, also includes fixed and random effects, which allows the errors to represent the deviation of observed data in relation to the expected values in the population. Consequently, the fixed regression parameters of the model represent the effect of covariates and are interpreted the same way as a generalized additive model for cross-sectional data. The following equation describes the structure of the GAMM:
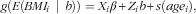
where *BMI*_*i*_ is the vector of BMI measured over time for participant *i*; *X*_*i*_ and *Z*_*i*_ are known covariate matrices of participant *i* including the intercept; *β* is the vector of fixed effects; and *b* is the vector of independent normally distributed random effects with zero mean. Specifically, a two-level model assuming a Gamma response variable distribution, logarithm link function (*g*(.)) and thin plate smoothing spline (*s*(.)) were fitted.

Given the gender differences of both the means and the trajectories of BMI, sex-specific models were estimated. Forward selection with the approximate Wald test was used for the inclusion of covariates. Subsequently, age, education level, race and year of data collection were kept in the model. We tested the following potential interactions: education level × race and education level × year of collection. The Wald test was also used to assess the significance of random effects in the intercept and the year of data collection and of the age smooth terms. Additionally, we calculated the standardized residuals to investigate possible deviations of the final model from the GAMM assumptions. Models were estimated by Penalized Quasi Likelihood implemented in the library mgcv^(^ ^23^ ^)^ of the software R, version 3·0·2^(^ ^24^ ^)^.

## Results

### Sample characteristics

The main differences between men and women were found in marital status, education level and per capita household income (Table 1). Comparing genders, men were more likely to be married and women were more likely to be separated or widowed (*P<*0·0001). The level of education was higher for women than men: 48 % and 34 % with college degrees, respectively; as was the per capita family income (*P<*0·0001). The average age at the first phase was 39·5 (sd 7·97) years for women and 38·6 (sd 8·23) years for men.

**Table 1:** Baseline characteristics of the population study according to gender. Pró-Saúde Study, Rio de Janeiro, Brazil, 1999–2006

	Men		Women		Total		
Characteristic	*n*	%	*n*	%	*n*	%	*P* value†
Age at first phase (years)							0·0297
20–30	161	14·4	151	10·6	312	12·2	
31–40	479	42·7	595	41·8	1074	42·2	
41–50	368	32·8	526	36·9	894	35·1	
51–60	96	8·6	132	9·2	228	9·0	
61–70	17	1·5	11	1·5	38	1·5	
Race							0·0024
White	596	52·9	792	55·0	1388	54·0	
*Pardo*	385	34·2	412	28·6	797	31·0	
Black	146	13·0	237	16·4	383	14·9	
Marital status							<0·0001
Married	793	70·4	788	54·7	1581	61·6	
Separated/widow	113	10·0	333	23·1	446	17·4	
Single	221	19·6	320	22·2	541	21·1	
Per capita family income (MW)							<0·0001
<3	379	33·6	339	23·5	718	28·0	
3–6	398	35·3	556	38·6	954	37·1	
>6	350	31·1	546	37·9	896	34·9	
Education							<0·0001
Elementary	277	24·6	247	17·1	524	20·4	
High school	464	41·2	496	34·4	960	37·4	
College	386	34·3	698	48·4	1084	42·2	
Parents’ education							0·7726
No education	66	5·9	99	6·9	165	6·4	
Primary	661	58·7	841	58·4	1502	58·5	
High school	238	21·1	299	20·7	537	20·9	
College	162	14·4	202	14·0	364	14·2	
Smoking habits							0·2488
Never smoked	650	57·7	878	60·9	1528	59·5	
Ex-smoker	199	17·7	234	16·2	433	16·9	
Smoker	278	24·7	329	22·8	607	23·6	

MW,minimum wage.

† *P* value of *χ* ^2^ test for independence.

### Observed and age-adjusted BMI trajectories

**Fig. 1:**
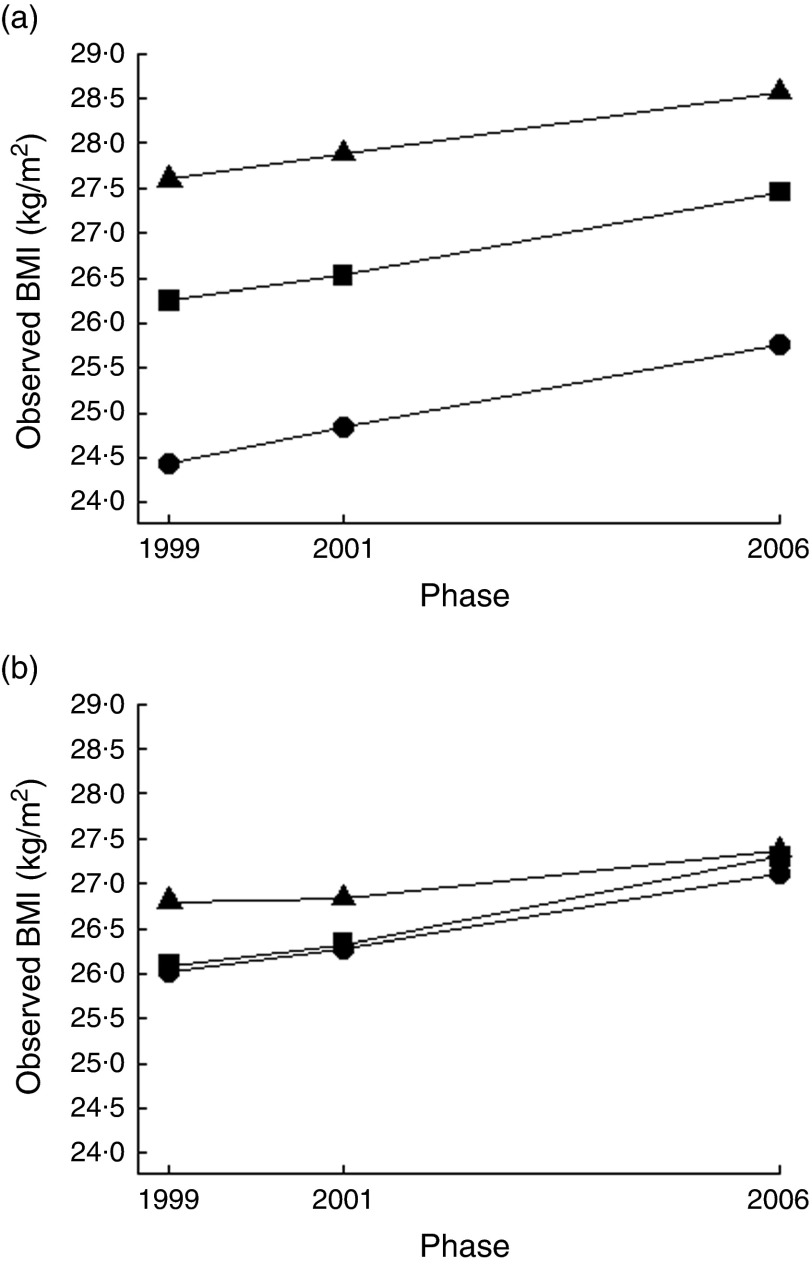
BMI trajectory of women (a) and men (b) by level of education (▲, elementary; ■, high school; ●, college). Pró-Saúde study, Rio de Janeiro, Brazil, 1999–2006

Figure 1 shows the trajectories over time of the observed mean BMI (crude), according to the level of education. Mean BMI increased between 1999 and 2006 for all education levels. Among women, those with the lowest educational level (elementary) had a mean BMI greater than the others in all phases of the study. Regardless of education level, women’s mean BMI trajectories showed an upward trend, but with no marked differences at the beginning and end of the assessment period. Among men, the mean BMI presented a distinct behaviour, with no differences between baseline values (1999), in contrast to that for women. In this unadjusted analysis, the effect of education on BMI varied over time. Between 1999 and 2001, mean BMI values among men with high school and college education were similar, and lower than those of men with an elementary education. The trend for BMI between 2001 and 2006 for groups with higher education (i.e. high school attainment) changed more markedly than that of men with elementary education, making the average BMI values similar by 2006.

**Fig. 2:**
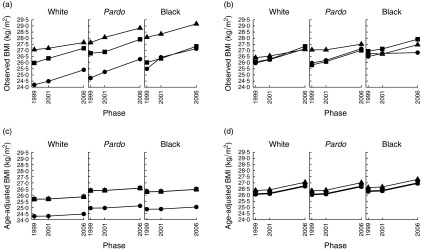
BMI trajectory of women (a, c) and men (b, d) by education level (▲, elementary; ■, high school; ●, college) and race: (a, b) observed mean BMI; (c, d) mean BMI adjusted for age. Pró-Saúde study, Rio de Janeiro, Brazil, 1999–2006

There was greater variation in mean BMI for women (24·2 to 28·1 kg/m^2^ in 1999, 25·4 to 29·2 kg/m^2^ in 2006) compared with men (25·8 to 27·1 kg/m^2^ in 1999, 26·8 to 27·9 kg/m^2^ in 2006; Fig. 2(a) and (b)). *Pardo* and Black women with less than college education had the largest mean BMI in all phases of data collection. The lowest mean BMI values and the most favourable trend were found in White women with a college degree (Fig. 2(a)).

After adjusting for the non-linear effect of age, the trends for women separated into four distinct trajectories. Black and *Pardo* women with elementary or high school education had the highest mean BMI in all phases. They were followed by White women with an elementary and high school education and these, in turn, were followed by Black and *Pardo* women with college education. White women with the highest education had the lowest mean BMI and the smallest increases over time (Fig. 2(c)).

Among men there was no clear distinction between baseline mean BMI values, except among *Pardo* men. Nor were there differences in changes in BMI across phases of the study. Even so, *Pardo* men with elementary education had the highest mean baseline BMI (27·05 kg/m^2^) and White men with high school education showed a higher increase (1·27 kg/m^2^ from 1999 to 2006; Fig. 2(b)). After controlling for age, men with an elementary school education, independently of race, were distinct from the other two levels of education. Of note, Black men with college education presented slightly higher values of mean BMI over time than their similarly educated White and *Pardo* peers (Fig. 2(d)). BMI values of Figs 1 and 2 are provided in the online supplementary material, Supplemental Tables 1 and 2.

### BMI trajectory (multiple analyses)

Table 2 presents the changes in BMI (exp(*β*)) estimated by GAMM. Positive values indicate an increase in BMI in relation to reference categories. In the modelling process, education (*P*<0·01), race (*P*=0·02) and smoking habits (*P*<0·01) contributed to explain the variance in the BMI trajectories of women. On the other hand, marital status, parental education and per capita family income were not statistically significant for women. The interaction terms between education and race (*P*=0·54) and between education and the year of data collection were also not statistically significant (*P*=0·96). Therefore, in the final model (M5) the covariates race and smoking habits were maintained and the estimated mean BMI for White women with college education, who were non-smokers, with ages centred on the mean of 1999, was 24·44 kg/m^2^ (reference).

**Table 2:** BMI variation (kg/m^2^) estimated by GAMM by gender. Pró-Saúde study, Rio de Janeiro, Brazil, 1999–2006

	Women					Men				
	M1	M2	M3	M4	M5	M1	M2	M3	M4	M5
Education										
Elementary *v*. college										
Estimate	1·11*	1·06*	1·07*	1·06*	1·06*	1·02	0·99	1·01	1·01	1·04**
95% CI	1·08, 1·14	1·03, 1·09	1·04, 1·10	1·03, 1·09	1·03, 1·09	1·00, 1·05	0·97, 1·02	0·99, 1·04	0·99, 1·04	1·01, 1·06
High school *v*. college										
Estimate	1·06*	1·06*	1·06*	1·06*	1·06*	1·00	1·00	1·00	1·00	1·01
95% CI	1·04, 1·08	1·04, 1·08	1·04, 1·08	1·04, 1·08	1·04, 1·08	0·98, 1·02	0·98, 1·02	0·98, 1·02	0·98, 1·02	0·99, 1·03
Race										
*Pardo v*. White										
Estimate				1·03**	1·03**				1·00	1·01
95% CI				1·01, 1·05	1·01, 1·05				0·98, 1·02	0·99, 1·03
Black *v*. White										
Estimate				1·02	1·02				1·00	1·02
95% CI				1·00, 1·05	1·00, 1·05				0·98, 1·04	0·99, 1·05

GAMM, generalized additive regression model with mixed effects. *P* value of the null hypothesis (*β*=0): *P<0·05, **P<0·001.

For women: M1=unadjusted; M2=M1+s(age); M3=M2+phase; M4=M3+race; M5=M4+smoke.

For men: M1=unadjusted; M2=M1+s(age); M3=M2+phase; M4=M3+race; M5=M4+smoke+marital status+income.

The estimated mean BMI (model M5) for White men with college education, average age, non-smoker, married and with an income greater than six minimum wages, was 25·55 kg/m^2^. Unlike for females, including race did not improve the fit of the model (*P*=0·78), but it was nevertheless kept in the final model (M5). In addition to education, age centred on the mean, along with race, smoking habits, marital status and per capita family income, were also included in the final model. No interactions of interest were statistically significant.

Compared with college-educated women, those with elementary or high school education gained more weight during the study period 1999 to 2006 (1·06 kg/m^2^ on average). There was an independent effect of race on increasing BMI (*P*=0·025). Compared with White women, Black women gained, on average, 1·02 kg/m^2^ and *Pardo* women gained 1·03 kg/m^2^ (Table 2). Overall, women and men gained slightly more than 1 kg/m^2^ from 1999 to 2006 (1·01 kg/m^2^ and 1·03 kg/m^2^, respectively).

Additionally, men with elementary and high school education gained more weight (1·04 kg/m^2^ and 1·01 kg/m^2^, respectively) than those with college education. However, unlike for women, there was no independent effect of race on increasing BMI. The smooth effect of age on the models for both genders was statistically significant (*P*<0·001; see Supplemental Figure 1, online supplementary material). Assessment of collinearity via the variance inflation factor suggested no dependency among key predictors (all variance inflation factors <2)^(^ ^25^ ^,^ ^26^ ^)^.

## Discussion

In the current study, men and women with the lowest level of education had the largest increases in BMI between 1999 and 2006. Race was independently associated with this trend only among women: Black and *Pardo* women with a lower level of education were the group with the most unfavourable trend. No interaction between education and race was found.

To the best of our knowledge, the present study is the first Brazilian one to investigate the simultaneous influence of education, race and gender on BMI trends in a cohort of working-age adults. The influence of race on health, together with indicators of socio-economic position, has rarely been studied in Brazil, where the history of race relations has its own specificities^(^ ^11^ ^–^ ^14^ ^)^.

Additionally, in the international literature, there are very few reports of the simultaneous study of socio-economic position, race and gender^(^ ^27^ ^–^ ^29^ ^)^. One of our key results – the inverse association between education and BMI – is consistent with those from studies conducted in Brazil and other countries^(^ ^5^ ^,^ ^8^ ^,^ ^28^ ^–^ ^30^ ^)^. Between 1986 and 2004, Clarke *et al*.^(^ ^28^ ^)^ observed in repeated cross-sectional analyses of 18–45-year-old US residents that BMI increased the most among women, racial minorities and those who had a lower educational level. In the Alameda County Study, Baltrus *et al*.^(^ ^31^ ^)^ confirmed these results during a 34-year follow-up; and, similar to our findings, reported that while Black women gained more weight than White women this was not the case for men.

Social inequalities in weight gain remain poorly understood. More favourable economic conditions clearly enable individuals to achieve and maintain ‘ideal body weight’. Access to information about healthy behaviour, access to lower-calorie foods (with regard to both price and availability), as well as the availability of time and accessible locations for physical activity directly depend on socio-economic conditions^(^ ^32^ ^,^ ^33^ ^)^.

However, contextual effects, beyond individual socio-economic resources, also contribute to the obesity epidemic. For example, the recent US recession coincided with a clear decline in the increase in female obesity in all income groups^(^ ^34^ ^)^, a finding previously reported in longitudinal studies in other countries^(^ ^35^ ^–^ ^38^ ^)^. According to these studies, when economic crises affect the population as a whole, a general decline in mean BMI or a deceleration in weight gain is observed.

In addition, in Brazil, as in many other countries, body perception and the desired body type differ by gender and socio-economic status. Women and individuals of higher socio-economic position seem more concerned about being overweight than men and groups with lower socio-economic position^(^ ^4^ ^,^ ^39^ ^)^. In a qualitative study of residents of Rio de Janeiro, Novaes^(^ ^40^ ^)^ compared the perceptions of women of higher education and income with those of less well educated and lower-income women. In both groups, beauty was considered an ‘article of prime necessity’. However, perceptions of what represented ‘beauty’ were not the same. In the case of the more affluent women, a thin body was the ideal, whereas ‘for [the] group of poor women, beauty is associated with generous or well delineated curves and not thinness’.

Also, the relationship between perceived exposure to racial discrimination and obesity has been documented in the USA and such discrimination has been hypothesized to be a form of chronic stress that can affect weight gain^(^ ^18^ ^)^. In our study, race presented an independent effect on women’s BMI trajectories. Therefore, the historical association between race, gender and health-damaging social exclusion warrants additional attention^(^ ^41^ ^–^ ^44^ ^)^, especially since neuroendocrine hormones due to chronic stress may also contribute to social inequalities in weight-gain trajectories^(^ ^10^ ^)^.

The comparison of results between studies conducted in different historical periods and geographical areas demands caution, because countries or population groups may be at different stages of the nutritional transition. Swinburn^(^ ^42^ ^)^ draws attention to the fact that the influence of social disparities in the trend of weight gain varies historically according to the exposure of different birth cohorts with different constraints and opportunities. In the last decade, Brazil achieved its lowest recorded level of income inequality^(^ ^43^ ^)^. Although the study population consists of civil servants, a group with relatively small variations in income, like persons in all social strata in the country, it was likely influenced by secular increases in the level of consumption of highly processed, consumption-ready foods, known to contribute to weight gain^(^ ^7^ ^)^.

In summary, our data point to the existence of complex mechanisms linking race, gender and socio-economic conditions to adverse BMI changes in a cohort of Brazilian civil servants. Our findings are consistent with those from studies conducted in the USA and UK indicating a higher risk for obesity among women not racially classified as White^(^ ^44^ ^–^ ^48^ ^)^. This differential risk for being overweight may be related to cultural differences in perceived ideal body size, limited knowledge regarding the role of energy imbalance in obesity risk, limited income to purchase healthier, more nutritious foods, and/or chronic psychological stress and its impact on eating behaviour and physical inactivity. The latter can be additionally impacted by adverse neighbourhood conditions. The differential effect of the nutritional transition on segments of the Brazilian population also merits increased research attention in efforts to understand the social patterning of obesity in the country.

In the present study, only individuals who participated in all three phases of data collection and who had valid information for all study variables were included in the analysis. Consequently, we considered how intermittent missing observations might bias our results (the overall attrition of participants was 18·7 % and 4·8 % in 2001 and 2006, respectively). Regarding potential loss to follow-up bias, our previous work with the Pró-Saúde cohort implicates a missing completely at random mechanism^(^ ^49^ ^)^. Specifically, we found no statistically significant difference between the average BMI of drop-outs in 2001 and those who finished the study (*P*=0·15). A complete case analysis treating the ignorable missing data produced wholly consistent findings^(^ ^50^ ^,^ ^51^ ^)^. To further corroborate these findings, an in-depth qualitative analysis on the causes of attrition revealed that retirement from the job was the principal reason. Moreover, attrition associated with a missing completely at random mechanism provides an unbiased estimation of effect^(^ ^52^ ^)^.

When comparing the participants of the 1999 census with the sample included in the study, no statistically significant differences in relation to the distribution of marital status, education, per capita family income or race were found.

The relatively short follow-up is also a potential study limitation, although education and race, our key exposures, are not highly subject to changes over time. One major strength of our study is that BMI values were based on physical measurements at each stage of data collection, rather than on self-reported weight and height.

The increasing prevalence of overweight and obesity and the complexity of factors responsible for their increase require the abandonment of simplistic explanations in favour of analyses focusing on factors located at different levels of the social hierarchy. The fact that an interaction term between education and race was not statistically significant could be due to sample size limitations. Notwithstanding, our results point out the importance of simultaneously considering the role of education, race and gender in the obesity pandemic, since for women, but not men, race showed an independent association with weight gain. Obesity is increasingly concentrated in socio-economically disadvantaged populations^(^ ^5^ ^,^ ^28^ ^)^. Thus, achieving better living conditions and reducing the social exclusion of these groups should be an essential part of anti-obesity efforts.

## Supplementary material

For supplementary material accompanying this paper visit http://dx.doi.org/10.1017/S1368980015001032. click here to view supplementary material
