# Supplementary material for: Social inequalities in BMI trajectories: 8-year follow-up of the Pró-Saúde study in Rio de Janeiro, Brazil
Source: Public Health Nutr. 2015 Apr 21;18(17):3183–91. doi: 10.1017/S1368980015001032 (PMC4642226; doi:10.1017/S1368980015001032)

2014

Supplement material

SOCIAL INEQUALITIES IN BMI TRAJECTORIES: 8 YEAR FOLLOW-UP OF THE PRÓ-SAÚDE STUDY IN RIO DE JANEIRO, BRAZIL

List of Tables

Table A - Observed mean of BMI (women and men) by levels of education. Pró-Saúde Study, Rio de Janeiro, Brazil. 1999–2006 2

Table B - Crude and age-adjusted mean of BMI (women and men) by education level and race. Pró-Saúde study, Rio de Janeiro, Brazil. 1999–2006 3

List of Figures

Figure A - Partial smooth effect of age in the final model with 95% bandwidth confidence intervals for both genders. Pró-Saúde study, Rio de Janeiro, Brasil. 1999 – 2006. (a) women; (b) men 4

Table A - Observed mean of BMI (women and men) by levels of education. Pró-Saúde Study, Rio de Janeiro, Brazil. 1999–2006

Phase	Education	Women	Men
1999	Elementary	27.59461	26.79620
2001	Elementary	27.88641	26.82916
2006	Elementary	28.56918	27.37056
1999	HighSchool	26.24721	26.09410
2001	HighSchool	26.52719	26.32351
2006	HighSchool	27.45155	27.29589
1999	College	24.43870	26.01318
2001	College	24.83741	26.27937
2006	College	25.77113	27.11711

Table B - Crude and age-adjusted mean of BMI (women and men) by education level and race. Pró-Saúde study, Rio de Janeiro, Brazil. 1999–2006

Phase	Education	Race	Women		Men	
			Crude	Age-adjusted	Crude	Age-adjusted
1999	Elementary	White	27.06540	25.66354	26.41037	26.38251
2001	Elementary	White	27.18077	25.67852	26.55058	26.43528
2006	Elementary	White	27.64637	25.85716	27.07807	27.04823
1999	HighSchool	White	25.97982	25.67135	26.06823	26.11274
2001	HighSchool	White	26.35359	25.68634	26.27553	26.16497
2006	HighSchool	White	27.17745	25.86503	27.33880	26.77165
1999	College	White	24.19534	24.27487	25.97315	26.05553
2001	College	White	24.48383	24.28904	26.25256	26.10765
2006	College	White	25.41636	24.45801	27.14040	26.71301
1999	Elementary	Brown	27.64312	26.36304	27.05266	26.35086
2001	Elementary	Brown	28.07010	26.37843	27.04969	26.40357
2006	Elementary	Brown	28.82845	26.56193	27.51258	27.01579
1999	HighSchool	Brown	26.77030	26.37106	25.81720	26.08142
2001	HighSchool	Brown	26.87973	26.38646	26.08830	26.13358
2006	HighSchool	Brown	27.90733	26.57002	27.00989	26.73954
1999	College	Brown	24.75021	24.93652	25.97280	26.02428
2001	College	Brown	25.24873	24.95107	26.19487	26.07633
2006	College	Brown	26.29163	25.12465	27.14336	26.68096
1999	Elementary	Black	28.07785	26.26759	26.75936	26.61475
2001	Elementary	Black	28.34204	26.28292	26.70482	26.66799
2006	Elementary	Black	29.16832	26.46576	27.46988	27.28634
1999	HighSchool	Black	26.01891	26.27558	26.93928	26.34261
2001	HighSchool	Black	26.34445	26.29092	27.12275	26.39530
2006	HighSchool	Black	27.33624	26.47382	27.91861	27.00733
1999	College	Black	25.50081	24.84623	26.50272	26.28490
2001	College	Black	26.44371	24.86074	26.75693	26.33747
2006	College	Black	27.14435	25.03368	26.82080	26.94816

Figure A - Partial smooth effect of age in the final model with 95% bandwidth confidence intervals for both genders. Pró-Saúde study, Rio de Janeiro, Brasil. 1999 – 2006. (a) women; (b) men

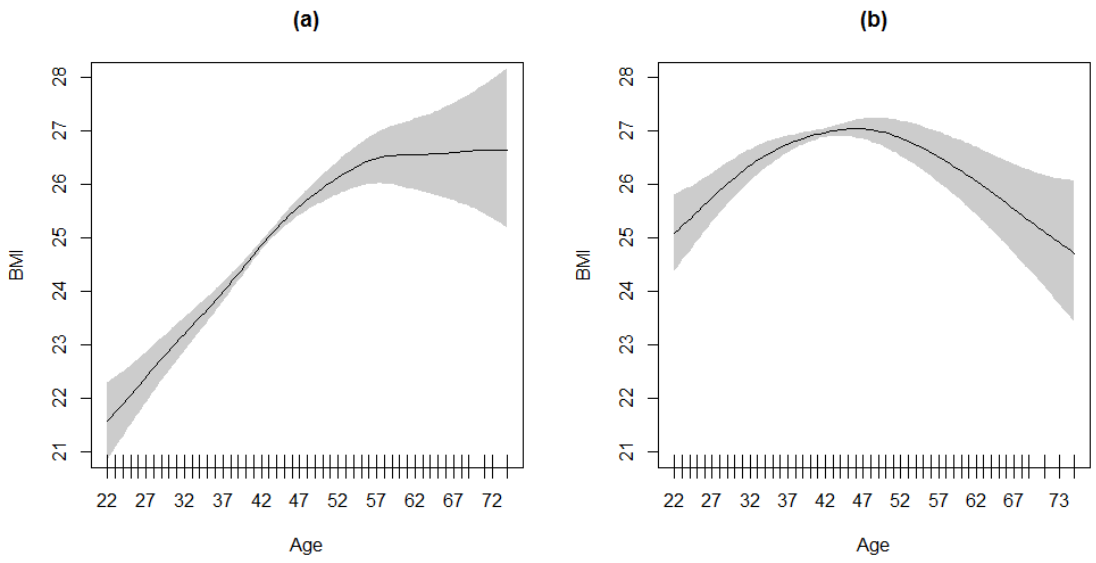

Supplement: Supplementary file 1 [file S1368980015001032sup001.pdf]
